# The Data Governance Act and the EU's move towards facilitating data sharing

**DOI:** 10.15252/msb.202110229

**Published:** 2021-03-23

**Authors:** Mahsa Shabani

**Affiliations:** ^1^ Metamedica Faculty of Law and Criminology Ghent University Ghent Belgium

**Keywords:** S&S: Ethics

## Abstract

The implementation of the EU General Data Protection Regulation (GDPR) has had significant impacts on biomedical research, often complicating data sharing among researchers. The recently announced proposal for a new EU Data Governance Act is a promising step towards facilitating data sharing, if it can interplay well with the GDPR.

## Graphical abstract

The EU General Data Protection Regulation (GDPR) has affected biomedical research, often complicating data sharing. The recently announced proposal for a new EU Data Governance Act, is a promising step towards facilitating data sharing.

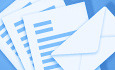

In an attempt to improve and increase data sharing in the EU and to optimize the re‐use of personal and non‐personal data, the European Commission has recently announced the proposal for a new EU Data Governance Act (https://ec.europa.eu/digital‐single‐market/en/news/proposal‐regulation‐european‐data‐governance‐data‐governance‐act). If approved, it will enable the creation and regulation of “secure spaces” where various types of data, including health data, can be shared and re‐used for both commercial and altruistic purposes, including scientific research. The Data Governance Act, within the framework of a European Strategy for Data, (https://ec.europa.eu/info/sites/info/files/communication‐european‐strategy‐dat‐19feb2020_en.pdf), would address some of the shortcomings and drawbacks of the current regulatory framework which holds back sharing and re‐using data for biomedical research purposes.

While the proposed Act would apply to all types of personal and non‐personal data, the increasing demand for sharing health data has most likely been a major rationale for this new legislation of data governance. Notably, sharing health and genetic data for scientific research entails an extra layer of complexity, owing to concerns over data protection and privacy when sharing sensitive personal data. *Vice versa*, there are also concerns in the scientific community over the negative impact of regulatory restrictions on sharing health data in data‐driven biomedical research. The pressing question here is how far the EU’s proposed legislative and policy framework can offset either concerns?

## Researchers and data access barriers

Databases from hospitals, laboratories, research institutes or patient registries have become a crucial resource for biomedical research, and the COVID‐19 pandemic has starkly highlighted the need for sharing health data for epidemiological research across borders. However, researchers face significant barriers accessing health and patient information owing to both an inadequate data‐sharing infrastructure and a restrictive regulatory framework, particularly when data are shared across institutions and countries (Bovenberg *et al,* 2020). Sharing sensitive health data in the EU must be compliant with the GDPR and data protection principles, notably purpose limitation, that is data can only be used for specific purposes. In the context of biomedical research, however, it is often crucial to be able to process data for multiple research purposes without requiring explicit consent for each downstream use.

Although many international and European funding organizations have stressed the importance of sharing data related to COVID‐19 (https://wellcome.org/coronavirus‐covid‐19/open‐data), the guidelines provided by data protection oversight bodies such as the European Data Protection Board (EDPB) (https://edpb.europa.eu/our‐work‐tools/our‐documents/ohjeet/guidelines‐032020‐processing‐data‐concerning‐health‐purpose_en) have not significantly relaxed the conditions regarding scientists’ access to and re‐use of such data. This article discusses how the proposed Data Governance Act may address some of the concerns related to the re‐use and sharing of health data for research purposes.

## Data‐sharing infrastructure and intermediaries

A major challenge for ensuring data protection and privacy is that sharing and analysing data in biomedical research often entails secondary uses or “further processing” by other scientists across the world. Adequate data governance mechanisms and secure data‐sharing platforms should be adopted to ensure that such further processing is compatible with the GDPR.

The proposed Act thus includes provisions about data‐sharing infrastructure, intermediaries and providers of such data‐sharing services. These can assist data holders, such as hospitals and research institutions, as well as data subjects—that is patients and research participants—with sharing their personal and non‐personal data for commercial and altruistic purposes. The activities of such data‐sharing services would be subject to oversight by competent authorities according to the proposed Act. Such platforms and other data‐sharing infrastructure should be compatible with the GDPR, namely ensuring that databases can only be accessed by authorized users for authorized purposes. This aspect is crucial in the context of biomedical research, because sensitive health data from patients and research participants require a higher level of protection to avoid potential abuse.

## European Data Altruism Consent: an opportunity for harmonizing consent models?

Importantly, the proposed Act also aims to introduce a uniform “European data altruism consent form” for altruistic data re‐use. It defines “data altruism” and “general interest” non‐strictly as “the consent by data subjects to process personal data pertaining to them, or permissions of other data holders to allow the use of their non‐personal data without seeking a reward, for purposes of general interest, such as scientific research purposes or improving public services”. The proposal further expounds that this consent should be considered as an additional legal certainty specially in the context of scientific research and contributes to additional transparency for data subjects.

Introducing a new consent model for data sharing can be both an opportunity and a challenge for biomedical researchers. At first sight, the proposed uniform consent model can be seen as yet another requirement for data sharing, on top of the other consent requirements for research with human subjects and for processing personal data. On the other hand, a uniform European consent for altruistic uses may become an opportunity to harmonize legislation across the EU Member States and thereby ease data sharing at least within the EU.

One example of how research would benefit from a uniform consent is the “1+ Million Genomes” initiative of the European Commission (https://ec.europa.eu/digital‐single‐market/en/european‐1‐million‐genomes‐initiative) to facilitate access to large‐scale genomic data across Europe. One of the major obstacles for this and other pan‐European projects is to coordinate regulatory frameworks, including consent requirement, among the partners. In this context, a harmonized approach to consent is particularly useful as the GDPR has left considerable room for implementation of the rules related to processing data for scientific research.

In fact, as oversight bodies have not supported a broad consent for scientific research (EDPB, 2020), the Member States have been left with the choice of either requiring specific consent for research or using other legal grounds such as public interest as a lawful basis for processing data. Depending on the relevant rules established by national regulations, the latter may remove the need for obtaining specific consent for data processing in scientific research. Although this option would seem to ease the regulatory burden for scientists, questions remain whether the principle of transparency and patients’ right to self‐determination are being adequately respected. Patients and research participants have a right to know how their data are being used and for which purposes. While the consent mechanism is admittedly far from perfect, it still provides some legal certainty that individuals have been informed about use of their data and exercised some level of control on that matter.

The proposed Act can address the identified caveat on using public interest as a basis for processing personal data. The proposed uniform consent should bring additional transparency for patients and research participants and give them a choice to opt in for sharing their data for altruistic purposes. In that sense, a consent for altruistic use can complement public interest as a legal basis for using data in research, which has been endorsed by the European Data Protection Supervisor (EDPS) in their preliminary opinion on the European Health Data Space (EDPS, 2020). While the success of the proposed uniform consent model depends on how this will be implemented in practice and how it will interplay with other consent requirements, the rationale behind it seems to align with the goal of empowerment of patients and participants.

## Individual control on data and the role of data cooperatives

The proposal has taken another step towards empowerment of individuals, by recognizing the role of intermediaries, such as data cooperatives, to support them on how their data are being shared. Such data cooperatives can assist to “negotiate terms and conditions for data processing before they consent, in making informed choices before consenting to data processing, and allowing for mechanisms to exchange views on data processing purposes and conditions that would best represent the interests of data subjects or legal persons”, as stated in the text of the proposed Act.

In recent years, the issue of empowerment of patients and research participants has been accentuated notably by patient groups who want better control over who can access their data and for which purposes. However, individual patients often lack the adequate expertise to fully understand the technicalities of the data‐sharing ecosystems. Adding this to the imbalance of power between data subjects and institutions who process data, the negotiating power of individuals regarding the terms and conditions of data use is significantly restricted. In reality, individuals are facing a so‐called consent dilemma or a take‐it‐or‐leave‐it option when consenting to the use and re‐use of their data. At times, this has led to frustration among individuals and patients’ groups who want to play a more active role in research.

In response, a limited number of data cooperatives, such as MIDATA (https://www.midata.coop/en/home/) and SALUS COOP (https://www.saluscoop.org), have been established. MIDATA, a Swiss non‐profit cooperative, acts as a “trustee for data collection and guarantees the sovereignty of citizens over the use of their data”. Participation in the data cooperative would enable citizens “to actively contribute to research as users of the platform by providing access to data sets and as cooperative members to control and develop the cooperative” (Hafen, 2019). Current examples include the use of the MIDATA platform to collect data from University Hospital in Bern on patients with a gastric bypass operation and University Hospital in Zurich for research projects patients suffering from multiple sclerosis (Hafen, 2019).

Notably, other emerging proposals such as the Data Union (https://thedataunion.eu) or the Data Dividend (Kelly, 2020) projects can, in principle, also facilitate data sharing by individuals for privately funded research. Indeed, this could enable health and DNA data markets for biomedical research sponsored by the private sector, where individuals share their data in exchange for financial incentives (Ahmed & Shabani, 2019). However, monetary incentives in health research have raised ethical and legal concerns, such as imposing undue influence on individuals and questioning the validity of their consent. The proposed Act has in fact not addressed the issues related to financial benefits for individuals who share their data for commercial purposes. This seems to be in line with the existing European approach which disfavours property rights on personal data and is catious to endorse monetizing personal data.

## Public trust on data‐sharing platforms

The proposal Act’s aim to ease the flow of data by providing a regulatory framework for data‐sharing infrastructure is a welcome step towards orchestrating data sharing for commercial and altruistic purposes. The Act should however interplay with relevant regulations such as the GDPR. It therefore remains to be seen whether future regulatory steps and implementation of the Act—if approved—would complement or complicate the current EU regulatory landscape for data sharing for research purposes.

The implementation of the GDPR has had significant impacts on biomedical research, mainly partly due to different consent requirements and legal grounds for data processing, which complicate data sharing among researchers. Furthermore, the experience so far has shown that more than mere adherence to data protection rules is needed to guarantee the success of data‐sharing programmes. The enormous amount of health and epidemiological data collected during the COVID‐19 pandemic is extremely valuable for biomedical research and has prompted the need to address all core elements of a robust governance framework for data. In particular, the tools and means for empowering individuals to keep control over how their data are being used and shared in a transparent environment should be integrated in future public health programmes for sharing health data. This way, we can hope that the upcoming data‐sharing programmes would succeed in wining public trust and support by the general public, which would greatly benefit biomedical research in turn.

## Conflict of interest

The author declares that she has no conflict of interest.
